# Asthma in Young Adults at High Risk for Allergies Is Traced Back to Immune and Microbiota Signatures in Early Childhood

**DOI:** 10.1111/cea.70283

**Published:** 2026-03-13

**Authors:** Ymke A. de Jong, Isabella Badolati, Ulrika Hellberg, Katarína Bankóová, Caroline Nilsson, Eva Sverremark‐Ekström

**Affiliations:** ^1^ Department of Molecular Biosciences, the Wenner‐Gren Institute Stockholm University Stockholm Sweden; ^2^ Department of Clinical Science and Education, Södersjukhuset Karolinska Institutet Stockholm Sweden; ^3^ Sachs' Children and Youth Hospital Stockholm Sweden

## Abstract

- Young adults with allergic asthma show altered immune phenotypic and transcriptomic features early in life.
- Key gut bacterial genera show distinct early‐life dynamics in 20‐year‐old asthmatics and non‐asthmatics.

**Abbreviations**
DC: dendritic cell
PBMC: peripheral blood mononuclear cell

To the Editor,

Allergic asthma is common in children and young adults and typically driven by IgE‐mediated immune responses to common environmental allergens. Family history of allergic disease is a well‐established risk factor [1], but a critical role for the early‐life gut microbiota has also been recognised, with links to an impaired immune tolerance and induction of pro‐allergic immune responses [2]. Several studies have reported associations between early‐life dysbiosis and asthma development during infancy and childhood [3], but long‐term consequences up to adulthood remain poorly understood.

Here, we investigated immune and gut microbiota profiles in 26 individuals from the BIAS cohort [1], recruited before birth between 1997 and 2000. To reduce the influence of genetic factors, all selected individuals had double heredity for allergy (i.e., two allergic parents, confirmed by medical history, positive skin prick test, and sIgE‐ab ≥ 0.35 kU_A_/L). At 20 years of age, 14 individuals were non‐asthmatic, while 12 had developed allergic asthma, defined by any of the following criteria within the last 12 months: problems with breathing (e.g., heavy breathing, chest tightness, wheeze), use of asthma medication, or a physician's diagnosis of asthma, together with IgE sensitization (sIgE ≥ 0.35 kU_A_/L to ≥ 1 of 16 allergens tested). We analysed blood samples (2 and 20 years) and faecal samples (1 and 4 weeks, 1 and 2 years) from the individuals, as well as maternal faecal samples (< 1 week from birth). Written informed consent was obtained at 20 years of age, while verbal parental consent was provided for earlier time points. For detailed methods, all demographic and clinical data, as well as additional experimental data not shown in Figure 1, see **online repository** at https://doi.org/10.5281/zenodo.18864389.

**FIGURE 1:**
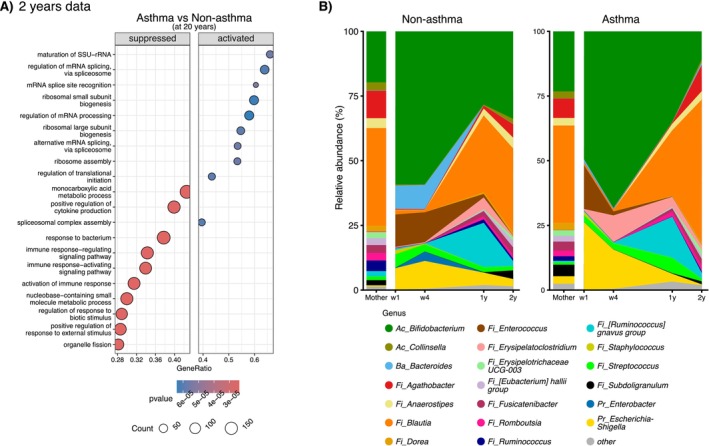
Early childhood differences in PBMC transcriptome and gut microbiota dynamics distinguish individuals with and without allergic asthma in young adulthood. In **A**), the bubble plot shows results of the gene set enrichment analysis performed on RNA sequencing data from PBMCs collected at 2 years, comparing individuals with allergic asthma by age 20 with those who did not develop asthma (Asthma and Non‐asthma, respectively). Upregulated pathways in asthmatics are shown on the right (activated), while downregulated pathways are shown on the left (suppressed). Colour of bubbles is based on *p*‐value, while size is based on gene counts. A cut‐off of *p* < 0.05 was used to filter the pathways. For the non‐asthmatic group, *n* = 8; for the asthmatic group, *n* = 9. In **B**), the average relative abundance (%) of gut bacteria at the genus level in the non‐asthmatic and asthmatic groups at different time points (1 week, 4 weeks, 1 year, and 2 years) and in their mothers. For the number of samples included at each time point, see **online repository**.

At 20 years, all individuals in the asthmatic group were IgE‐sensitized, compared to 8 of 14 non‐asthmatics. Food allergy and rhinitis were more common in asthmatics, whereas no differences between groups were observed earlier in life. The groups did not differ in sex or most early‐life characteristics, although C‐section delivery was more common among asthmatics (5 vs. 1 among non‐asthmatics).

Peripheral blood mononuclear cells (PBMCs) from the 2‐ and 20‐year time points were analysed by flow cytometry and RNA sequencing. At 20 years, overall immune phenotypes showed substantial inter‐individual variability and no clear separation between asthmatics and non‐asthmatics, possibly explained by the strong and comparable genetic predisposition for allergy in both groups and the use of asthma medication among asthmatic individuals. At 2 years, the differences were more pronounced; individuals who later developed asthma exhibited significantly higher frequencies of FcεRI^+^ dendritic cells (DCs) and lower frequencies of PD‐L1^+^ DCs, with similar trends observed in monocytes. These results are in line with previous research, reporting higher FcεRI expression on DCs from allergic subjects, linked to IgE levels and allergen responsiveness [4], while PD‐L1 is an inhibitory marker regulating T cell activation [5], and lower levels in the asthmatic group may indicate decreased ability in dampening T cells.

Transcriptomic profiling also revealed more extensive between‐group differences at 2 years than at 20 years (216 versus 60 significant differentially expressed genes), supporting the notion that early‐life immune alterations may precede asthma development, whereas later immune dysregulation may be confounded and masked by other factors. Several genes previously linked to asthma and allergic inflammation, such as *CX3CR1*, *IFITM3*, *FAM129A*, *DUSP8*, *FFAR1*, *TNFAIP8L2* (TIPE2) and *APOE*, were differentially expressed between the asthmatic and non‐asthmatic groups. Gene set enrichment analysis showed suppression of immune response‐related pathways, including responses to microbial stimuli, and enrichment in RNA processing pathways, at both time points but particularly at 2 years (Figure 1A). Notably, RNA splicing and non‐coding RNAs have recently been implicated in asthma pathogenesis [6], suggesting that aberrant RNA processing may contribute to altered gene expression profiles.

Plasma proteomic analysis at both 20‐ and 2‐years of age using the Olink platform showed no major differences in asthma‐associated cytokines between the groups, consistent with previous findings suggesting that these markers have a strong genetic contribution [7]. Levels of the barrier‐associated factors uteroglobin and LPS‐binding protein (LBP) were also comparable.

Analysis of the gut microbiota by 16S rRNA sequencing indicated no significant differences in α‐ or β‐diversity between the groups, with temporal shifts primarily driven by age. However, non‐asthmatics displayed a tendency towards higher richness and evenness at 1 year. Distinct dynamics were observed over time in the abundance of bacterial taxa (Figure 1B). At the genus level, *Bifidobacterium*, associated with asthma‐protective effects [3], was more abundant in non‐asthmatics at 1 week and 2 years, whereas the opposite pattern was seen from 4 weeks to 1 year, suggesting that timing and species composition of the bifidobacteria may influence asthma risk. *Escherichia‐Shigella* and *Erysipelatoclostridium*, previously linked to childhood asthma [3], were enriched in future asthmatics during the first month of life, while non‐asthmatics showed higher abundance of *Bacteroides*, reported to protect against asthma development [3]. Although this partly aligns with differences in delivery mode, as *Bacteroides* has been shown to be reduced after C‐section [8], it does not fully explain our findings, given that 7 asthmatic participants were delivered vaginally. Also, approximately 20% amplicon sequence variants overlapped between mothers and infants at 1 week, suggesting that maternal microbiota may also shape asthma risk.

Predictive gut microbiota functional profiling revealed upregulation of purine and pyrimidine degradation pathways in asthmatics at 1 week, whereas at 2 years, non‐asthmatics showed an enrichment in pathways associated with short‐chain fatty acid production, which is linked to protection against allergic disease [9].

Strengths of our study include the longitudinal follow‐up of a well‐characterized cohort up to 20 years of age, particularly regarding allergic disease and heredity, and the availability of multiple sample types across several time points. Limitations include the small sample size and the lack of PBMCs and plasma before 2 years, which reduced statistical power and prevented cross‐dataset correlation analyses. The limited sample size also restricted adjustment for potential confounders. Finally, the absence of a comparison group without allergic heredity limits interpretability.

In summary, we show that allergic asthma in young adulthood can be traced back to early‐life immune and gut microbiota signatures, even in individuals with high hereditary risk. These findings suggest that modulatory interventions during the early ‘window of opportunity’ may shape long‐term immune trajectories and impact asthma susceptibility.

## Author Contributions

Study design: E.S.‐E., C.N., Y.A.J., I.B. and U.H. Clinical data collection: C.N. and U.H. Experimental work: I.B. and Y.A.J. Data analysis: Y.A.J., I.B., U.H., K.B., E.S.‐E. and C.N. Drafting of manuscript: Y.A.J., I.B. and U.H. All authors critically reviewed the manuscript before submission.

## Funding

This study was financially supported by The Swedish Research Council (2020–01839 and 2023–02616), The Swedish Heart‐Lung Foundation, The Asthma‐ and Allergy Association's research foundation, The Cancer‐ and Allergy Foundation, Hesselmans Foundation, Stiftelsen Samariten, The Golden Jubilee Memorial, Föreningen Mjölkdroppen, The Swedish Order of Freemasons, and Stockholm University.

## Ethics Statement

The study was approved by the Regional Ethic Committee in Stockholm (Dnr; 0–2 years −75/97, 117/97, 20 years −2019/02034). The participants gave written consent at 20 years of age, while for earlier time points their parents provided informed verbal consent. At the time of initiation of the study, no written documentation of participants' approval was required, which was agreed to by the Ethics Committee. Research was conducted following the 1964 Helsinki Declaration and its subsequent revisions.

## Conflicts of Interest

C.N. reports grants to institution from Aimmune Therapeutics, a Nestlé Company, material for IgE analyses from Thermofisher used in academic studies, and lecture fees from MEDA ALK, Thermofisher and GSK. E.S.‐E. has received honoraria for lectures and a grant for another research project from BioGaia AB. No conflicts of interest reported from the other authors.

## Data Availability

Supporting data can be found at https://doi.org/10.5281/zenodo.18864389 and will be publicly available upon acceptance. The 16S rRNA sequencing data have been deposited at the NCBI Sequence Read Archive (BioProject ID: PRJNA1338459). The RNA sequencing data that were generated for this study and all other raw data supporting our findings are available upon request from the corresponding author.
